# An Insight into Survivin in Relevance to Hematological, Biochemical and Genetic Characteristics in Tobacco Chewers with Oral Squamous Cell Carcinoma

**DOI:** 10.3390/cells12101444

**Published:** 2023-05-22

**Authors:** Susanna Theophilus Yesupatham, C. D. Dayanand, S. M. Azeem Mohiyuddin, M. L. Harendra Kumar

**Affiliations:** 1 Department of Biochemistry, Sri Devaraj Urs Academy of Higher Education and Research, Tamaka, Kolar 563103, Karnataka, India; susanna020682@gmail.com; 2 Allied Health and Basic Sciences, Sri Devaraj Urs Academy of Higher Education and Research, Tamaka, Kolar 563103, Karnataka, India; 3 Department of Otorhinolaryngology and Head and Neck Surgery, Sri Devaraj Urs Academy of Higher Education and Research, Tamaka, Kolar 563103, Karnataka, India; 4 Department of Pathology, Shridevi Institute of Medical Sciences and Research Hospital, Sira Road, Tumakuru 572106, Karnataka, India

**Keywords:** survivin, *BIRC5*, NLR, LMR, PLR, OSCC, nicotine

## Abstract

Background: Survivin is an inhibitor of apoptosis protein (IAP), encoded by the Baculoviral IAP Repeat Containing 5 (*BIRC5*) gene located on q arm (25.3) on chromosome 17. It is expressed in various human cancers and involved in tumor resistance to radiation and chemotherapy. The genetic analysis of the *BIRC5* gene and its protein survivin levels in buccal tissue related to oral squamous cell carcinoma (OSCC) in South Indian tobacco chewers has not been studied. Hence, the study was designed to quantify survivin in buccal tissue and its association with pretreatment hematological parameters and to analyze the *BIRC5* gene sequence. Method: In a single centric case control study, buccal tissue survivin levels were measured by ELISA. A total of 189 study subjects were categorized into Group 1 (n = 63) habitual tobacco chewers with OSCC, Group 2 (n = 63) habitual tobacco chewers without OSCC, and Group 3 (n = 63) healthy subjects as control. Retrospective hematological data were collected from Group 1 subjects and statistically analyzed. The *BIRC5* gene was sequenced and data were analyzed using a bioinformatics tool. Results: Survivin protein mean ± SD in Group 1 was (1670.9 ± 796.21 pg/mL), in Group 2 it was (1096.02 ± 346.17 pg/mL), and in Group 3 it was (397.5 ± 96.1 pg/mL) with significance (*p* < 0.001). Survivin levels showed significance with cut-off levels of absolute monocyte count (AMC), neutrophil/lymphocyte ratio (NLR), and lymphocyte/monocyte ratio (LMR) at (*p* = 0.001). The unique variants found only in OSCC patients were T → G in the promoter region, G → C in exon 3, C → A, A → G, G → T, T → G, A → C, G → A in exon 4, C → A, G → T, G → C in the exon 5 region. Conclusions: The tissue survivin level increased in OSCC patients compared to controls; pretreatment AMC, LMR, and NLR may serve as add-on markers along with survivin to measure the progression of OSCC. Unique mutations in the promoter and exons 3–5 were observed in sequence analysis and were associated with survivin concentrations.

## 1. Introduction

Oral cancer is one of the most frequent malignant tumors worldwide, with major predominance seen in the populations of Southeast Asia and India [1,2]. In India, the oral cavity is one of the five leading sites of cancer, and 90% of these tumors are squamous cell carcinoma (SCC) [3,4]. The high incidence of oral cancer in India is due to addiction to tobacco chewing and smoking. A variety of tobacco habits are prevalent in India and they differ from region to region [5,6].

**Figure 1:**
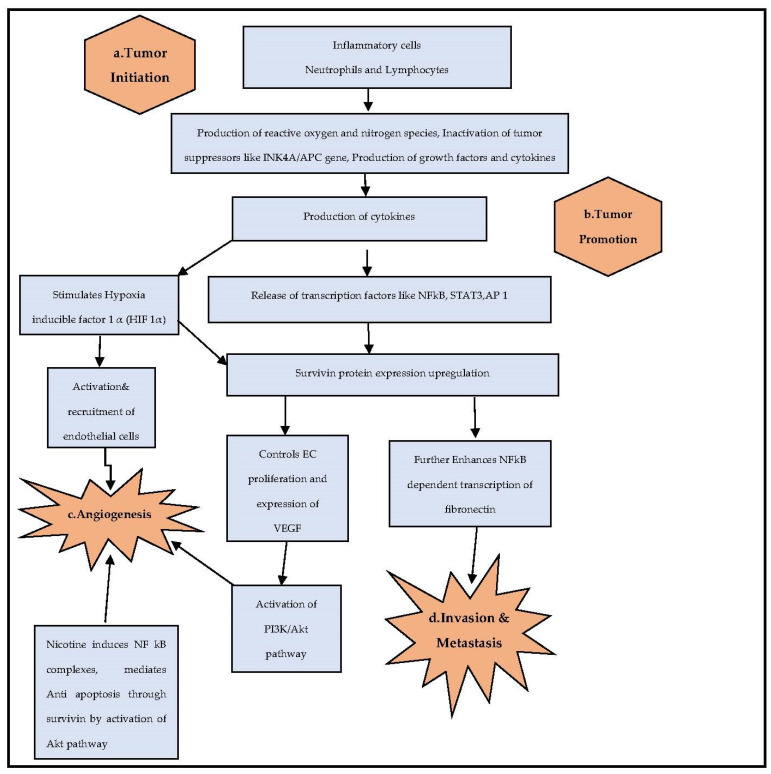
Role of inflammatory cells and survivin protein in tumorigenesis [11,12,13,23,24]. **a**. Tumor initiation: Inflammatory cells neutrophils, lymphocytes, and macrophages aid in tumor initiation through secretion of multiple cytokines and chemokines; release of reactive oxygen and nitrogen species damage the cellular DNA and cause genomic instability; inactivation of tumor-suppressor genes such as cyclin-dependent kinase inhibitor protein (INK4A) and the adenomatous polyposis coli gene (APC); and overexpression of growth factors and cytokines. **b**. Tumor progression: Cytokines release transcription factors such as nuclear factor kappa B subunit (NFkB), signal transducer and activator of transcription 3 (STAT3), activator protein 1 (AP 1); nicotine upregulates the expression of survivin by inducing NF-kβ complexes and through the Akt-dependent pathway brings about cell survival, inhibition of apoptosis, and progression of the tumor. **c**. Angiogenesis: Cytokines stimulate the production of hypoxia inducible factor alpha (HIFα) that activates and recruits endothelial cells; survivin induces the proliferation of endothelial cells and expression of vascular endothelial growth factor (VEFG). VEGF activates the PI3K and Akt pathways; nicotine is also known to activate the Akt pathway, all of which stimulate angiogenesis. **d**. Invasion and metastasis: Survivin enhances NFkB-dependent transcription of fibronectin and promotes tumor cell invasion and metastasis.

Chronic inflammation and cancer are interrelated. Inflammation is a chief component in the modulation of the tumor microenvironment [7,8]. The initiation, development, invasion, and metastasis of the neoplastic process are accompanied by inflammation and immune response. Furthermore, the survival of a tumor cell is based on sustained cellular proliferation and suppression of apoptosis regulation. Recently, the hematological parameters that are routinely measured, such as red cell distribution width (RDW), white blood cell count (WBC), platelet count (PLTC), and lymphocyte/monocyte ratio (LMR), are gaining attention as diagnostic and prognostic markers for gastric cancer, pancreatic adenocarcinoma, and non-small-cell lung cancer [9]. Studies have reported that tumor development and progression is induced by inflammatory response mediated through pro-inflammatory cytokines and nuclear factor kappa B (NF-kβ), signal transduction and activation of transcription factor 3 (STAT3) pathways [10,11,12,13] (Figure 1).

Oral smokeless tobacco consumed in betel quid containing betel leaf, areca nut, calcium hydroxide (slaked lime), and tobacco is a major cause of oral squamous cell carcinoma in the Indian subcontinent, parts of Southeast Asia, China, and Taiwan [14]. Areca nut and tobacco has been declared a known human carcinogen by an IARC Expert Group [14,15]. In India, tobacco chewing accounts for nearly 50% of cancers of the oral cavity in men and over 90% in women [16].

Tobacco contains tobacco-specific nitrosamines (TSNs), namely, 4-(nitrosomethylamino)-1-(3-pyridyl)-1-butanone (NNK) and N’-nitrosonornicotine (NNN). Animal studies have also shown that NNK and NNN in the tobacco products cause tumors of the oral cavity. NNK, NNN, and their metabolites covalently bind with deoxyribonucleic acid (DNA) in the cells, forming DNA adducts. These adducts are responsible for critical mutations involved in DNA replication [17].

A study by Quanri Jin et al. investigated the effects of the tobacco components nicotine and its related carcinogen 4-(methylnitrosamino)-1-(3-pyridyl)-1-butanone (NNK) on survivin expression in normal human bronchial epithelial (NHBE) cells and examined the role of survivin in the malignant transformation of normal human bronchial epithelial (HBE) cells induced by these components. They observed that survivin messenger RNA (mRNA) expression was detected in 41% of bronchial brush specimens from heavy smokers. Nicotine and NNK increased survivin mRNA and protein expression levels in primary cultured NHBE cells and immortalized HBE cells. Nicotine and NNK stimulated the Akt–mammalian target of rapamycin (mTOR) pathway in NHBE cells, leading to increased de novo synthesis of survivin protein [18].

Another study by Piyali Dasgupta et al. reported that nicotine inhibits apoptosis induced by the chemotherapeutic drugs gemcitabine, cisplatin, and taxol by upregulating XIAP (X-linked inhibitor of apoptosis protein) and survivin; they further inferred that the induction of survivin protein in non-small-cell lung cancer cells was through the Akt pathway [19].

Nicotine is a natural tobacco alkaloid chemically known as 3-(1-methyl-2-pyrrolidinyl) pyridine identified in oral and lung cancer as evidenced from in vitro and in vivo models [20,21]. Nicotine is reported to have an inhibitory effect on apoptosis through the phosphorylation of antiapoptotic protein, inducing NF-kβ complexes and the Akt-dependent pathway in lung cancer cells, which are known to reduce the action of chemotherapeutic drugs by upregulating survivin expression [19,22,23,24] (Figure 1). Despite this, peripheral blood-based biomarkers of tissue-based survivin in tobacco chewers with OSCC have not been studied so far. Therefore, we attempted to evaluate the pre-treatment clinical value of hematological parameters from a routine blood test and observed the association of these parameters with tissue survivin levels in habitual tobacco chewers with OSCC.

Survivin protein is the smallest member of the inhibitor of apoptosis protein (IAP) family, known to be involved in cell cycle regulation, inhibition of apoptosis, and microtubule stability [25]. It is a 16.5 kDa monomer protein with 142 amino acid residues coded by the Baculoviral IAP Repeat Containing 5 (BIRC5) gene located on human chromosome 17q25.3 that spans 14.7 kb at the telomeric end [26].

Survivin overexpression has been reported in human malignancies, such as breast (90.2%) [27,28], liver (87%) [29,30], bladder (57.8%) [31,32], stomach (68%) [33,34], oral (>75%) [35,36], and hematological malignancies (68%) [37], and its expression levels were observed to correlate with tumor stage or response to therapy [38]. The research findings suggested that survivin may serve as a biomarker in cancerous conditions. Our earlier research mentioned the quantitative estimation of tissue survivin and caspase 3 among the population addicted to tobacco chewing, in tobacco chewers with oral cancer [39].

The expression of survivin in a cell takes place in a cell cycle-regulated manner, and the peak levels of survivin are observed in the G2/M phase of the cell cycle [40,41,42]. In their study, Srivastava K et al. described the regulation of survivin expression to be transcriptionally controlled involving cell cycle-dependent elements (CDEs) and cell cycle homology regions (CHRs) located on the survivin gene promoter region [43]. Population-based studies on survivin expression by Nassar et al., Ryan BM et al., and Khan S et al. indicated survivin gene polymorphisms to be associated with human cancers [44,45,46]. In addition, -31G/C polymorphism was reported as a common mutation observed in cancer cell lines, resulting in the overexpression of survivin [47]. A study by Chan H Han et al. on survivin promoter polymorphisms (-1547 A/G,-644C/T,-625C/G,-241C/T and -31G/C) and their association with the age of onset of ovarian cancer observed that -625C/G was persistently involved in ovarian cancer compared to other polymorphisms [48]. The genetic variant -31G/C in the survivin promoter region has been identified as associated with the overexpression of survivin and increased risk for lung cancer and esophageal cancer [49,50]. The above studies were based on meta-analysis and the Restriction Fragment Length Polymorphism (RFLP) technique. Gene sequencing and nucleotide variants analysis, however, will help identify unique mutations and SNPs in the promoter regions in cancers in relation to the presence of causative factors.

A study by Xun C et al. on the mutation profile of oral squamous cell carcinoma cells reported that the C > T transition variant was the commonest mutation observed in the CpG dinucleotide region of the genes in tongue cancer cell lines [51]. They also analyzed mutations in a spectrum of 20 cancer genes and reported mutations in oral cancer were quite different from those in other tumors. Single nucleotide polymorphism (SNP) at the TP53 exon 4 codon 72 was observed as a risk factor for development of cancer, including OSCC, since variants in the coding regions of the genes can affect the expressed protein of the gene functionally [52]. A study by R. Ralhan et al. reported that the potentially malignant and cancerous lesions in the oral cavity harboring missense mutations of TP53 at codons 126,136, and 174 showed accumulation of p53 protein and its circulating antibodies [53]. However, as far as our knowledge on *BIRC5*/survivin is concerned and per the literature search, there are no studies available on the complete gene sequencing of the coding regions of the *BIRC5* gene in tobacco chewers with OSCC.

The cumulative information on survivin’s role in carcinogenesis in relation to OSCC is limited, and the genetic evidence in humans is not available in association with oral cancer, particularly OSCC caused by tobacco. Therefore, we aimed to quantify the survivin protein and to sequence and analyze the *BIRC5* gene sequence data to understand the molecular basis of the role of survivin in OSCC, which will be helpful in the future in community screening for the detection and management strategies of OSCC.

## 2. Materials and Methods

### 2.1. Patients

The study design was a single centric case control consisting of a total of 189 participants enrolled by the Dept. of Otorhinolaryngology and Head and Neck Surgery, R.L. Jalappa Hospital and Research Center, a rural referral tertiary care hospital attached to the Sri Devaraj Urs Medical College, Tamaka, Kolar, Karnataka, India. Individuals aged 30–65 years were included in the study and categorized into Group I (*n* = 63), habitual tobacco chewers with clinically proven OSCC cases; Group 2 (*n* = 63), habitual tobacco chewers without OSCC; and Group 3 (*n* = 63) healthy subjects as controls. The study was approved by the Central Ethics Committee of the University in a vide No. SDUAHER/KLR/CEC/33/2018-19 dated 14 May 2018. The inclusion criteria for subjects in the study were known habitual tobacco chewers with OSCC, habitual tobacco chewers without OSCC, and healthy controls with no history of addiction to tobacco or alcohol. Patients with a history of onco-surgery or neoadjuvant chemotherapy or with an immunodeficiency, recurrent or chronic ulcerative lesions of the oral cavity such as pemphigus/Behcet’s syndrome, or who had undergone radiotherapy, were excluded from the study.

### 2.2. Methods

#### 2.2.1. Sample Collection

Buccal tissue specimens resected from the buccal mucosal from Group 1 OSCC cases were collected in a phosphate buffered saline (PBS) (pH 7.2–7.4). Tissue homogenate was prepared. After the participants rinsed their mouths thoroughly with distilled water, buccal cell scrapings were obtained from the Group 2 and Group 3 subjects with soft buccal brushes supplied from the Puregene Buccal Cell Kit from Qiagen, Germantown, MD, USA, as per the procedure described [54]. The cell viability and count were determined using trypan blue stain and counted using a hemocytometer.

#### 2.2.2. Human Survivin Assay

The supernatant obtained from the buccal tissue homogenate in the above step was used for estimation of human survivin. The sandwich ELISA method was used as per the kit procedure (K12-5528, kinesis Dx, Los Angeles, CA, USA). The assay range of human survivin was 31.259 to 200 pg/mL.

#### 2.2.3. Pretreatment Hematological Parameters

The retrospective pretreatment hematological secondary data of OSCC patients were obtained from the medical records section. The hematological parameters comprised the counts of total white blood cells (WBCs), neutrophils, lymphocytes, monocytes, platelets, red blood cells (RBCs), packed cell volume (PCV), and hemoglobin concentration using a cell counter (SYSMEX XN, Kobe, Japan). From this data the neutrophil/lymphocyte ratio (NLR), lymphocyte/monocyte ratio (LMR), and platelet lymphocyte ratio (PLR) were calculated and presented.

#### 2.2.4. Evidence of Tissue Survivin Protein by Immunohistochemistry

The tissue content of survivin was determined by using 4 µm thickness sections from 10% formalin-fixed paraffin embedded tissues. Antigen retrieval was performed with citrate buffer pH 9 using a decloaker, primary mouse monoclonal antibodies against survivin (IHC 668, GenomeMe, GenomeMe Lab Inc., Richmond, BC, Canada), and a secondary antibody linked to horseradish peroxidase, which specifically binds to the primary antibody. Chromogenic substrate 3,3′ diaminobenzidine tetrahydrochloride (DAB) was then added and counterstained with hematoxylin. A strong expression of survivin in a colon adenocarcinoma specimen was used as positive control, and the colon adenocarcinoma specimen treated only with the secondary antibody was used as a negative control for determination of survivin by the immunohistochemistry technique.

#### 2.2.5. BIRC5/Survivin Gene Analysis

Isolation and purification of DNA from tumor tissue and buccal cell scrapings from study group subjects were carried out as per the literature supplied by Qiagen Germany (DNeasy Blood and Tissue Kit Cat. No. 69504, Qiagen, Hilden, Germany). The purified DNA sample was quantified by a NanoDrop device (ND-1000 UV-VIS Spectrophotometer, Thermo Fisher Scientizfic, Waltham, MA, USA). Suitable forward and reverse primer pairs specific to the sequence of promoter and exons of the *BIRC5* gene were designed using the Primer 3 tool for amplification and they were also validated by primer stats. The specificity of the selected primer pairs against the exon and promoter region was checked using Primer Blast and is presented in the Table 1.

##### PCR Amplification of BIRC5 Gene

The *BIRC5* promoter and exons of the gene were amplified using specific forward and reverse primers, as indicated in Table 1 at optimum PCR conditions. The total reaction volume of the PCR mix was 30 μL, using EmeraldAmp PCR master mix (Clontech Takara Cellartis Cat. no. RR310A). The components of the PCR mix were nuclease-free water 10 μL, Emerald master mix buffer 15.5 μL, forward primer 1.5 μL, reverse primer 1.5 μL, DNA template 1.5 μL. The amplification reactions were carried out in a thermo cycler with a sequence of initial denaturation at 95 °C for 5 min, 35 cycles of denaturation at 95 °C for 30 s, annealing temperature for 30 s, initial extension at 72 °C for 1 min, and final extension for 72 °C for 5 min. The reaction of the promoter and each exon allowed for extension of all amplicons.

##### Analysis of PCR Ampliconson Agarose Gel Electrophoresis

The aliquots (3 μL) of PCR amplicons of each exon and promoter region of the *BIRC5* gene were applied to 2% agarose gel in Tris-acetate-ethyl-diamine tetra acetate (TAE) buffer along with 100 bp DNA ladder to check the right product size and its migration on horizontal gel electrophoresis. The electrophoresis was performed in TAE buffer at a voltage of 120 volts and a current of 140 milliamps for 45 min, The amplicons in the gel run were visualized using ethidium bromide stain in the BIOBEE UV Digital Gel documentation system. The amplicons were extracted from the agarose gel using the LivgenGel extraction kit (Livgen Cat. No. MP011). The PCR products were purified with GeneJET PCR Purification Kit from Thermo Fisher Scientific, Waltham, MA, USA.

##### BIRC5 Gene Sequencing and Sequence Analysis

The amplified promoter region and exons of the *BIRC5* gene from representative samples were sequenced by Sanger’s dideoxy method using the Big Dye Terminator v3.1 cycle sequencing kit as per manufacturer’s instructions (Applied Biosystems, Foster City, CA, USA) and analyzed using ABI-3500 Genetic Analyzer (Thermo Fisher Scientific, Waltham, MA, USA). The chromatograms were viewed and analyzed using FinchTv and Applied Biosystems DNA Sequencing Analysis Software version 5.1. The FASTA format of the nucleotide sequences were submitted to the National Center for Biotechnology Information Basic Local Alignment search tool for nucleotides (NCBI BLAST-N), the reference nucleotide sequence was also mined from NCBI (NC_000017.11). The BIRC5 gene exon and promoter variants were further analyzed using the bioinformatics tool PredictSNP2 (single nucleotide polymorphism), a unified integration platform for accurately evaluating single nucleotide variation or polymorphism and its effects by exploiting different characteristics of the variants in a distinct genomic region considering PredictSNP1 prediction output [55].

### 2.3. Statistical Analysis

The research results were statistically analyzed using a licensed version of SPSS Software version 22 (IBM, New York, NY, USA). The normality check was conducted using the Shapiro–Wilk test with skewness. Continuous data were represented as mean and standard deviation (SD). Univariate analysis of blood parameters with tumor stage (T1–T2) and (T3–T4), node involvement (N_0_ and N_1_–N_3_), and levels of survivin protein in tumor tissues was conducted using the independent *t* test; *p* < 0.05 was considered statistically significant. Selected blood parameters were included for the multiple linear regression model to determine their association with survivin protein levels in OSCC patients; *p* < 0.05 was considered statistically significant. Furthermore, in order to determine the prognostic importance of survivin in relation to WBC counts, NLR, LMR and PLR, the study group patients (Group 1) were subdivided into 2 sub-groups, such as a Group with high and a Group with low values of each hematologic marker on the basis of cut-off values selected from published data on cancer patients and were compared with the survivin levels. The cut-off values of the parameters were WBC = ≥7.9 × 10^9^/L and <7.9 × 10^9^/L, ANC = ≥4.9 × 10^9^/L and <4.9 × 10^9^/L, ALC = >1.980 × 10^9^/L and ≤1.980 × 10^9^/L, AMC = ≥0.50 × 10^9^/L and <0.50 × 10^9^/L, NLR = ≥2.39 and <2.39, LMR = >3.22 and ≤3.22, PLR = >110.6 and ≤110.6 [56,57,58]. *BIRC5* gene sequence analysis data were tabulated in a Microsoft Excel sheet, and the data were presented under the headings: (a) single nucleotide variation, (b) number of subjects, (c) frequency of occurrence of variation in each group (expressed as a percentage (%)), and (d) average concentration of tissue survivin in each group.

## 3. Results

### 3.1. Patient Characteristic Details

The study participants came from a rural background with poor nutritional habits and lower socioeconomic status. However, the majority of the oral cancer patients visiting the hospital for treatment purposes had advanced disease. As per the American Joint Committee on Cancer Staging Manual, 18 patients (28.6%) were T4N1, 11 patients (17.5%) were T3N1, 05 patients (7.9%) were each T2N0, T2N1, and T3N0, 04 patients (6.3%) were T4N0, and 03 patients (4.8%) each with T2N2b, T3N2b, and T4N2a. All patients with oral cancer had Grade 1 well-differentiated squamous cell carcinoma.

### 3.2. Analysis of Tissue Survivin Data

The data obtained were tabulated as mean ± SD. There was a significant elevation of tissue survivin levels in OSCC patients (1670.9 ± 796.21 pg/mL) compared to the levels in buccal cell samples of habitual tobacco chewers (1096.02 ± 346.17) and healthy controls (397.5 ± 96.1 pg/mL), with *p* value < 0.001, as shown in Table 2.

### 3.3. Statistical Representation of Pretreatment Hematological Parameters

**Figure 2:**
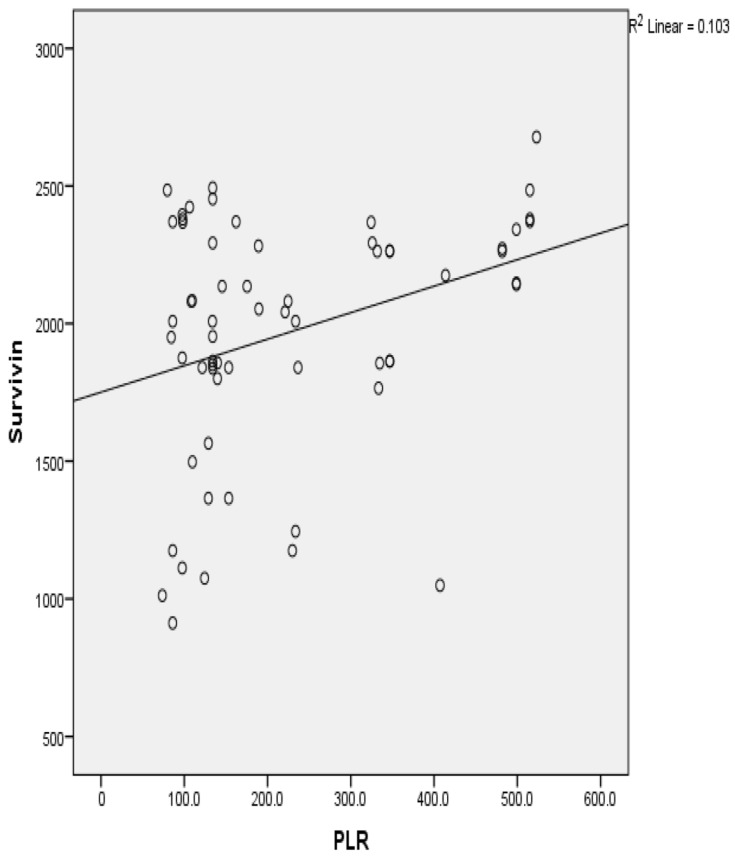
Correlation of platelet/lymphocyte ratio with survivin levels in OSCC patients.

The baseline hematological parameters in OSCC patients (n = 63) from the secondary source of data are presented as mean and standard deviation (SD), wherein hemoglobin levels, red blood cell (RBC) counts, packed cell volume (PCV), mean corpuscular volume (MCV), mean corpuscular hemoglobin concentration (MCHC), red cell distribution (RDW), white blood cell (WBC) count, absolute neutrophil count (ANC), absolute lymphocyte count (ALC), absolute monocyte count (AMC), platelet count (PLT) values were presented and the neutrophil/lymphocyte ratio (NLR), platelet/lymphocyte ratio (PLR), and lymphocyte/monocyte ratio (LMR) were calculated as shown in Table 3. For the same parameters, a univariate analysis was conducted in relation to tissue survivin levels and presented as shown in Table 4 and evinced a platelet/lymphocyte ratio significance (*p* = 0.01) when correlated with tissue survivin levels; the same is shown in Figure 2.

Univariate analysis of hematology parameters with different stages of tumor development ranging from T1–T2 and T3–T4 and with involvement of lymph node N0 (noninvolvement of lymph node) and N1–N3 (involvement of lymph nodes). The analysis showed significance (*p* = 0.008) with RDW with respect to tumor stage and node involvement, as seen in Table 5.

**Figure 3:**
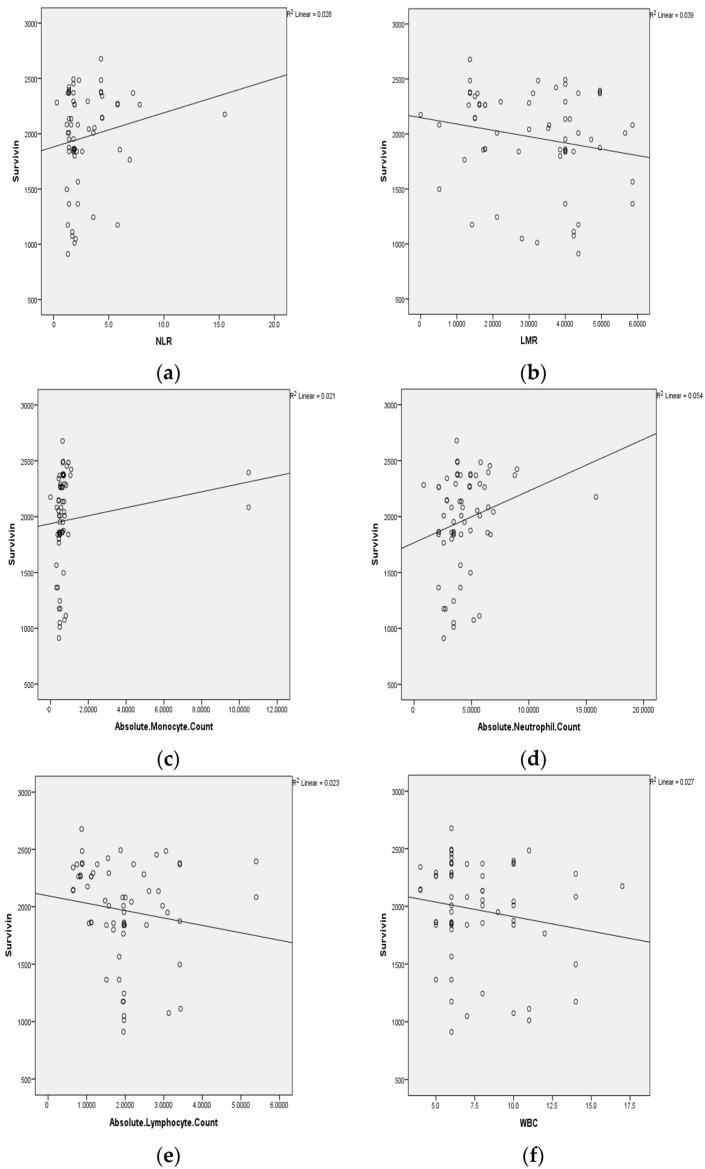
(**a**) No significant correlation of neutrophil/lymphocyte ratio with survivin levels, (**b**) significant correlation of lymphocyte/ monocyte ratio with survivin levels, (**c**) significant correlation of absolute monocyte count with survivin levels, (**d**) no significant correlation of absolute neutrophil count with survivin levels, (**e**) no significant correlation of absolute lymphocyte count with survivin levels, (**f**) no significant correlation of white blood cell count with survivin levels determined by multiple linear regression analysis in OSCC patients.

Multiple linear regression analysis of the hematology parameter with tissue survivin showed significance with AMC (*p* = 0.045 *) and LMR (*p* = 0.025 *), as presented in Table 6 and Figure 3. An independent *t* test comparing the mean values of tissue survivin with hematological variables WBC, ANC, ALC, AMC, NLR, PLR, and LMR in oral squamous cell carcinoma patients showed significance with AMC, NLR, and LMR (*p* = 0.001), whereas other parameters were not significant, as shown in Table 7.

### 3.4. Demonstration of Survivin Protein by Immunohistochemistry Technique

**Figure 4:**
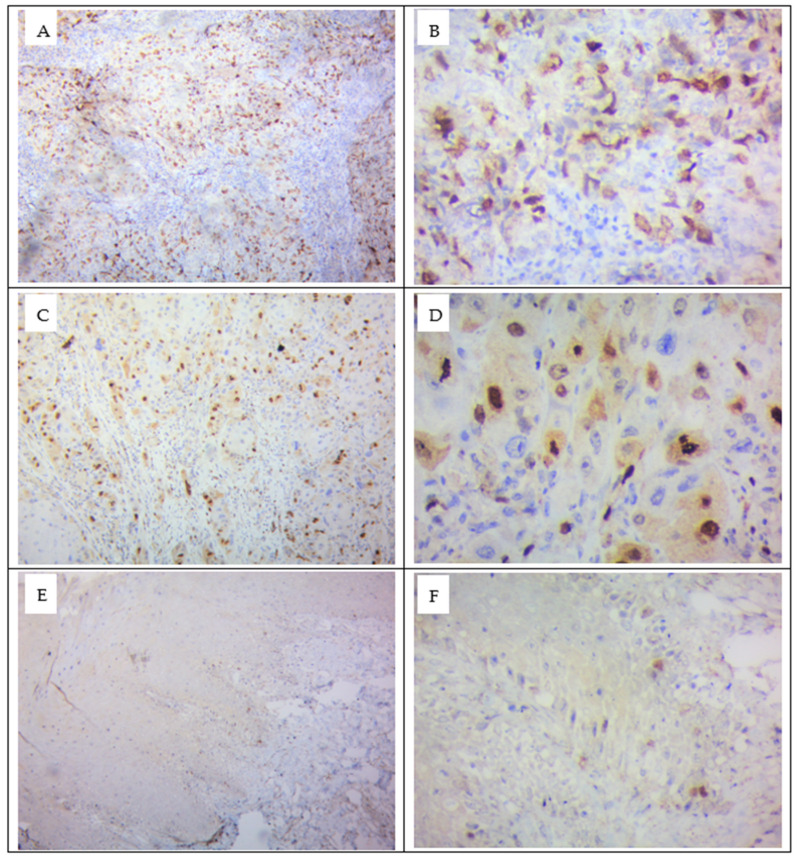
Photomicrographs of immunohistochemical staining for survivin in oral squamous cell carcinoma tissues (**A**–**D**) and normal buccal mucosa (**E**,**F**). Immunohistochemical staining shows positive staining in brown. Photographs captured with a digital camera at 100× and 400× magnification under identical conditions.

Tissue samples from primary oral squamous cell carcinoma patients (n = 24) were collected from the Department of Otorhinolaryngology and Head and Neck Surgery. Normal buccal mucosa tissues (n = 5) were obtained from non-tobacco users during extraction of impacted molars upon obtaining informed consent. Tissue samples from primary OSCC and from normal buccal mucosa were fixed in formalin and embedded in paraffin blocks in the Pathology Laboratory of RL Jalappa Hospital and Research center, Tamaka, Kolar, and used for detection of survivin by immunohistochemical technique. The expressed tissue survivin protein was scored, based on the scoring criteria followed in a method described by Jane et al.; according to these criteria, less than 5% of the cell staining indicated 0 (negative staining), between 5% and 25% of cell staining indicated +1 (weak staining), 25–50% of the cell staining indicated +2 (moderate staining), and >50% of the cell staining indicated +3 (strong staining) [59]. Four samples (17%) showed +3 strong staining, eight samples (33%) demonstrated +2 moderate staining, ten samples (42%) showed +1 weak staining, and two samples (8%) showed negative staining for the survivin antigen. Furthermore, all the samples exhibited nuclear staining and three samples with strong staining for survivin exhibited both nuclear and cytoplasmic staining. Control samples showed 0–5% cells stained for survivin protein and they were scored as negative even though minimal staining in the cells was evidenced (Figure 4).

### 3.5. BIRC5 Gene Sequence Analysis

Forty samples were selected based on the levels of high tissue survivin concentrations from the study subjects of each group for promoter sequence analysis, specifically, 20 samples from OSCC patients, 10 buccal cell samples from habitual tobacco chewers without OSCC, and 10 buccal cell samples from healthy controls. These were screened with *BIRC5* promoter region sequence analysis to identify known single nucleotide variations. Seventy samples were selected based the mean and +SD and mean and –SD of survivin concentrations for exons 1–5. Sequence analysis of BIRC5, specifically, 30 samples from OSCC patients, 20 buccal cell samples from habitual tobacco chewers without OSCC, and 20 buccal cell samples from healthy controls.

From the above samples we found from promoter and exons of *BIRC5* sequence analysis data, the promoter region comprising 397 bp revealed a total of eight single nucleotide variants. Accordingly, the notable single nucleotide variants determined were the G → T variant in 35% of OSCC cases, 30% in habitual tobacco chewers without OSCC, and 30% in healthy subjects, whereas T → G variants were observed only in 5% of OSCC patients, a unique G → A variant was observed only in 30% of habitual tobacco chewers. A G → C variant was observed in 15% of OSCC patients and 30% of healthy controls, and the same was not seen in the habitual tobacco chewers group. However, A → T, C → T, C → G, and G → T variations were observed in all three groups and their frequency and percentage of occurrence are depicted in Table 8. The above promoter variants of the study subject were observed with an average tissue survivin level, and it was found that the G → T variant was observed with 2228.58 pg/mL in OSCC patients, 1104.12 pg/mL in habitual tobacco chewers, and 443.65 pg/mL in healthy subjects. The T → G variant was observed with 2082.88 pg/mL in OSCC patients, the G → C variant observed with 2194.29 pg/mL in OSCC patients and 437.12 pg/mL in healthy subjects (Table 8).

In the exon 1 region comprising 175 bp, two notable single nucleotide variants (C → A and G → C) were observed in exon 1 at 5′ untranslated region (UTR) in OSCC patients, tobacco chewers without OSCC, and healthy subjects; the C → A variant was observed in 33.33% of OSCC patients, 5% of tobacco chewers without OSCC, and 5% of healthy subjects. On the other hand, G → C was observed in 86.67% of OSCC patients, 50% of tobacco chewers without OSCC, and 45% of healthy subjects (Table 8). The average tissue survivin levels were found in the C → A variant with 1649.3 pg/mL in OSCC patients, 1197.3 pg/mL in habitual tobacco chewers without OSCC, and 457.82 pg/mL in healthy subjects. The G → C variant was noted with 1632.77 pg/mL in OSCC patients, 1045.56 pg/mL in tobacco chewers without OSCC, and 398.22 pg/mL in healthy subjects.

Four variants—G → T, C → G, T → G, C → T—were noted only in OSCC patients with 26.67%, 6.67%, 6.67% and 3.33%, respectively. The average tissue survivin levels observed were 2015.04 pg/mL in the G → T variant, 2110.49 pg/mL in the C → G variant, 2427.92 pg/mL in the T → G variant, and 1565.42 pg/mL in the C → T variant. The G → A variant was found in 4% (1400.68 pg/mL) of OSCC patients and in 10% (450.04 pg/mL) of the healthy subjects and was not found in habitual tobacco chewers.

In the exon 2 region comprising 110 bp, a total of four single nucleotide variants (G → C, G → A, C → T, A → C) were observed, of which each G → C and G → A variant was found in 3.33% (1074.94 pg/mL) of OSCC patients, and the same variants were present in 15% (432.24 pg/mL) and 10% (428.65 pg/mL), respectively, of healthy subjects. The C → T variant was present only in 5% (428.65 pg/mL) of healthy subjects, A → C was present in 5% (303.97 pg/mL) of habitual tobacco chewers, and 5% (432.24 pg/mL) of healthy subjects (Table 8).

In the exon 3 region comprising 69 bp, two nucleotide variants were observed in exon 3, of which C → T was found only in 10% (720.05 pg/mL) of habitual tobacco chewers without OSCC and the G → C variant was found only in 3.33% (2485.07 pg/mL) of OSCC patients (Table 8). 

In the exon 4 region comprising 118 bp in the BIRC5 gene, 11 variants were observed, of which 6 variants (C → A, A → G, G → T, T → G, A → C, and G → A) were found in 3.33% (2370.77 pg/mL), 6.67 % (2272.69 pg/mL), 10% (1711.07 pg/mL), 3.33% (1850.2 pg/mL), 6.67% (1807.81 pg/mL), and 3.33% (1765.42 pg/mL), respectively, only in OSCC patients. The G → C and T → A variants were found only in 15% (1266.55 pg/mL) and 10% (1157.7 pg/mL), respectively, of tobacco chewers without OSCC, and G → C was found in 5% (422.81 pg/mL) and T → A in 5% (227.01 pg/mL) of healthy subjects. The C → T variant was present in 5% (422.81 pg/mL) of only healthy subjects. The A → T variant was present in all three groups, in 6.67% (1691.5 pg/mL) of OSCC patients, 5% (1415 pg/mL) of tobacco chewers without OSCC, and 10% (406.12 pg/mL) of healthy subjects. The T → C variant was present in 3.33% (1850.2 pg/mL) of OSCC patients and 10% (1310 pg/mL) of tobacco chewers without OSCC (Table 8). 

In the exon 5 region comprising 2171 bp, nine variants (A → C, C → A, T → A, G → A, C → T, G → T, T → G, G → C, A → T) were observed in the exon 5 region of the BIRC5 gene, of which three variants, C → A in 3.33% (1850.2 pg/mL), G → T in 6.67 % (1542.09 pg/mL) and G → C in 3.33% (1850.2 pg/mL), were observed only in OSCC patients. Three variants, T → A, G → A and C → T, were observed in all the groups as shown in Table 8. 

Exonic sequence analysis of the BIRC5 gene revealed a larger number of OSCC patient samples predominantly showing variations in the promoter region, 5′ untranslated region, and exon 1 region, which were predicted to be deleterious in nature; the exon variants observed were subjected to the PredictSNP1 tool to determine any alteration in the amino acid sequence in the survivin primary protein structure.

## 4. Discussion

The present study focused on determining the interrelation between tobacco consumption and the levels of survivin and its association with the hematological parameters of the participants. To the best of our knowledge, per the available literature, the current study is the first to evaluate the association of tissue survivin levels with pretreatment levels of the hematological parameters in chronic tobacco chewers with OSCC. Our study revealed that the hemoglobin concentration in all OSCC patients was below the normal cut-off range of 12–15 gm% in females and 17 gm% in males. Malnourishment seen in cancer patients results in anemia [60,61]. The increase in RBC count seen in cancer patients may be hypoxia-driven and related to inflammation in bone marrow tissue [62].

Studies have reported that the systemic inflammation triggered by cancer alters the number of circulating immune cells such as neutrophils and lymphocytes [56]. Moreover, the peripheral blood cell counts of cancer patients reflect tumor progression and were found to have a prognostic value, according to a number of published reports connecting hematological markers with cancer prognosis. Therefore, hematological parameters in the blood test serve as the criteria for differentiating cancer patients and healthy individuals [56,63,64].

Upon univariate analysis of the hematological parameters with tumor staging, nodal involvement, and tissue survivin protein concentration, we found that with regards to tumor staging, absolute lymphocyte count and absolute platelet count were found to be elevated among T1–T2 stage patients, compared to T3–T4 stage. With respect to node involvement, the RDW was found to be significantly high among N1–N3 patients compared to N0 individuals; the elevated RDW signifies inflammation and poor nutritional status in these patients. However, we did not find any other hematological parameter to be associated with tissue survivin protein levels. This was in contrast to a study by Yuzhen Luo et al. wherein a significant elevation in RDW in 127 cases of urothelial carcinoma of the bladder was seen compared to control subjects, but their results did not show any significance with respect to tumor stage or lymph node involvement. Upon multiple linear regression analysis, we found tissue survivin protein levels to be significantly associated with the absolute monocyte count and lymphocyte/monocyte ratio, and none of the other hematological parameters had any significant association with the tissue levels of survivin protein in OSCC patients [65].

On comparison of tissue survivin levels with hematological parameters, cut-off values having prognostic significance such as white cell count, absolute neutrophil count, absolute lymphocyte count, absolute monocyte count, neutrophil/lymphocyte ratio, lymphocyte/monocyte ratio, and platelet/lymphocyte ratio.

We observed that 38 (60%) OSCC patients had an absolute monocyte count of (≥0.500 × 10^9^/L) significantly associated with increasing tissue survivin levels. The neutrophil/lymphocyte ratio was found to be significantly higher and associated with tissue survivin levels in 21 (33%) OSCC patients, and the lymphocyte/monocyte ratio was found to be significantly lower and associated with tissue survivin levels in 30 (48%) OSCC patients (Table 6). The above findings generated a clue about survivin influence on increasing the neutrophil/monocyte ratio and decreasing the lymphocyte/monocyte ratio, indicating its contribution to the poor prognosis in OSCC patients.

It is well established that host response plays a vital role in the determination of the biological behavior of tumors. Studies have recently reported an elevated neutrophil/lymphocyte ratio (NLR) to correlate with the aggressive biological behavior in various malignancies including head and neck tumors. Nakashima et al. reported that a higher NLR of >2.4 was associated with advanced OSCC and poor response to chemotherapy. Phulari et al. observed a mean NLR of 2.84 in OSCC compared to healthy controls and suggested NLR could be a surrogate marker for the aggressive behavior of oral squamous cell carcinoma [66,67]. Similarly, our study also observed a higher NLR of ≥2.91 in 50 (79%) oral squamous cell carcinoma patients with tumor stage T3–T4 and an NLR of ≥2.67 in 13 (26%) patients with tumor stages T1–T2, the higher NLR (≥2.91) indicated the tumor stage progress of OSCC. However, patients were not followed up to assess the treatment outcome on the NLR in OSCC.

The mechanism by which inhibitor of apoptosis protein survivin alters the neutrophil, lymphocyte, and monocyte counts is still unclear; however, several in vitro experimental studies on cell culture, using bone marrow cells from cancer patients, observed inflammatory cytokines to extend the survival of neutrophils by delaying apoptosis [68,69].

Survivin expression was found to be induced in terminally differentiated neutrophils by cytokines such as granulocyte/macrophage colony stimulating factor (GM-CSF) and granulocyte CSF (G-CSF), which prolong the neutrophil lifespan, suggesting the importance of survivin in blocking apoptosis in neutrophils in a cell cycle-independent manner [70]. The NLR compares the inflammatory response to the immune response of the host, the interplay of neutrophils and lymphocytes in tumor initiation and their indirect effect on the tissue survivin expression ultimately leading to tumor promotion, angiogenesis, and metastasis, as shown in Figure 1.

Research studies have demonstrated that a low lymphocyte/monocyte ratio is associated with a worse overall survival (OS) in patients with bladder cancer and pancreatic cancer [71,72]. Circulating monocytes infiltrate the tumor and differentiate into tumor-associated macrophages. Tumor-associated macrophages are known to suppress adaptive immunity through secretion of variety of chemokines and cytokines such as tumor necrosis factor-α, interleukin (IL)-1, IL-6, and IL-10 to promote tumor growth, angiogenesis, invasion, and migration [73,74]. This is shown to be associated with poor prognosis in cancers [75,76,77].

In their study, Tsai et al. observed a decreased lymphocyte count and an increase in peripheral monocytes and the neutrophil/lymphocyte ratio with the advancement of clinical stage in oral cancer patients and suggested that pretreatment peripheral monocyte count could serve as an independent predictor of worse prognosis in oral cancer patients [78]. In our study we observed that around 60% of the patients with OSCC had a significantly higher monocyte count than the cut-off value and this correlated with increasing tissue survivin levels, but we did not find any significant variation with respect to tumor staging or node involvement.

Lymphocytes play an important role in adaptive immune response. Through immune surveillance they have been found to eliminate early tumor cells by cytotoxic cell death and the production of cytokines. Studies have reported that infiltrating lymphocytes indicate the generation of an effective antitumor cellular immune response [79,80]. However, in established tumors the adaptive immune response is suppressed through pathways such as inhibition of dendritic cell differentiation and activation, and infiltration of regulatory T cells [81]. A low peripheral lymphocyte count thus indicates a poorer lymphocyte-mediated immune response to tumor and suggests poor prognosis [82,83]. Studies have reported the LMR as an independent prognostic factor in patients with bladder cancer, esophageal cancer, and malignant pleural mesothelioma [84,85,86,87,88,89]. A study by Fardeela BIN-ALEE et al. on the evaluation of lymphocyte apoptosis in patients with oral cancer reported the levels of helper (Th) cells that promote anti-tumor immune response to be significantly higher in oral cancer patients than in hepatic cancer patients. Furthermore, they found an increase in B cells and cytotoxic T cells to eliminate tumor cells by secreting cytokines such as TNF and IFNγ [90]. However, the percentage of Th cells was slightly lower in highly metastatic N3 tumors in OC patients, which may have escaped the T cell-mediated immune response mechanism by the adaptation of primary tumor antigens [91].

They also found a high level of (Bax) Bcl-2 associated X, a pro-apoptotic protein, and B-cell lymphoma-2 (Bcl-2), an anti-apoptotic protein ratio, in oral cancer stage IV patients. Tumor size, lymph node involvement, and the Bax/Bcl-2 ratio were also higher in advance-stage tumors, which suggested that Bax/Bcl-2 ratio levels are associated with OSCC aggressiveness [92]. In our study also, we observed that 43 (68%) of the OSCC patients had peripheral lymphocyte levels <1.980, though not statistically significant, which suggests a poor prognosis in these patients, and the majority of our patients had advanced tumors. However, ascertaining the poor prognosis in relation to the lowered peripheral lymphocyte count and impact of adaptive response demands follow-up of the patients, as it is a lacunae in the current study.

The intracellular expression of survivin as demonstrated by immunohistochemistry indicated that all the tissue samples of tobacco chewers with OSCC exhibited nuclear staining, and three of these samples with strong staining for survivin exhibited both nuclear and cytoplasmic staining. Study reports on the localization of survivin are controversial; a study by Chiao-Ying Lin et al. observed cytoplasmic expression of survivin in the areca nut chewing Taiwan population, and nuclear survivin expression was shown to be associated with poor prognosis in non-small-cell lung cancer and esophageal squamous cell cancer [93,94]. Studies have reported that cytoplasmic survivin regulates the action of caspases, and nuclear survivin is part of the CPC, related to cellular division [95,96]. In the current study we found a higher concentration of survivin and higher nuclear staining in tobacco chewers with OSCC compared to control samples. The finding in the study indicated tobacco as a contributing factor to increasing survivin concentration, and its predominant nuclear staining of survivin can be considered as a predictor factor for the malignant progression of the cells in habitual tobacco chewers with OSCC [39].

Furthermore, it has been reported in previous studies that the regulation of survivin expression occurs at multiple levels, which include transcription, translation, and posttranslational modification [97]. A group of transcription factors, such as Sp1, NF-kB, STAT3, E2F1, and Kruppel-like factor 5 (KLF5), have been demonstrated to bind with the survivin promoter and enhance survivin expression in tumors, whereas the p53, forkhead box O3 (FOXO3), and early growth response 1 transcription factor (Egr-1) inhibit expression of survivin in cells [97,98]. Beyond transcriptional and translational regulation, recent reports have revealed that survivin also undergoes various posttranslational modifications that include phosphorylation, acetylation, and ubiquitination. Phosphorylation is required for survivin stabilization, subcellular trafficking, and biological activation. Phosphorylation on Thr34 prevents ubiquitination-induced survivin destruction [99].

An in vitro study by Ming Li et al. demonstrated that the natural compound xanthohumol decreased survivin phosphorylation at Thr34 through inhibition of Akt-Wee1-CDK1 signaling, which in turn facilitated E3 ligase survivin ubiquitination and degradation in squamous cell carcinoma cells [100].

To date, per our knowledge and a literature survey, this is the first study on the *BIRC5* gene polymorphism reporting in chronic tobacco and alcohol users with and without OSCC among the rural South Indian population. Currently, the relevant genetic data on exonic mutations of the BIRC5 gene and the biological effect of survivin function are limited; therefore, we made an attempt to discuss the BIRC5 gene polymorphism on the basis of a bioinformatics approach.

The *BIRC5*/survivin gene is functionally involved in the upregulation of the G2/M checkpoint of the cell cycle at the mitotic spindle apparatus. The *BIRC5* gene plays a key role in preserving the transformation of normal cells into cancer cells. It also promotes the angiogenesis and proliferation of these cancer cells. Therefore, genetic variations in the survivin/BIRC5 gene may cause an unusual survivin expression and contribute to carcinogenesis through interference with the functional domains of survivin.

The promoter region of the BIRC5 gene contains the binding motif for cell cycle-dependent elements/cell cycle homology region (CDE/CHR). Several studies conducted in the past have mainly focused on the polymorphisms in the promoter region of the BIRC5 gene such as –31 G/C, a single nucleotide polymorphism at the promoter region (SNP: −31 G/C) that was shown to alter the binding sites for CDE/CHR, thus inducing survivin overexpression in these cancers. These polymorphisms were studied in breast cancer, prostate cancer, esophageal and colorectal cancer. The promoter polymorphism was found to be associated with increased risk for development of breast cancer [101,102,103,104]. However, there is paucity of studies that have evaluated the variations in the promoter region of the *BIRC5* gene in OSCC patients. In our study we found that the T → G variation was found only in the OSCC patient samples, and this may be the reason for the twofold increased survivin levels in comparison to tobacco chewers and an increase in survivin levels of 4.5-fold in comparison to healthy subjects. A unique G → A variation observed in tobacco chewers alone predicted as deleterious by the prediction tools may be the reason for the twofold rise in survivin concentration in tobacco chewers in comparison to healthy controls. A → T, C → T, C → G, and G → T variations that were observed in all three groups may suggest that the subjects in the habitual tobacco chewers group and healthy subjects group possessing these mutations are at increased risk in the presence of epigenetic factors for developing OSCC. In our study the G → T variation was present at highest % in all three groups with the highest average concentration of survivin compared to the other variations observed in the promoter region. It is further evidenced from the immunohistochemistry staining of OSCC and healthy subjects (normal buccal mucosa) that 12 (50%) of the OSCC samples showed strong to moderate staining for survivin and 2 (40%) of the healthy buccal mucosa tissues showed 5% staining for survivin.

In the exon 1 region variants, in a combined output generated by the bioinformatics prediction tool PredictSNP, T → G, C → A, C → T, and C → G variants found in the 5′ untranslated region (5′UTR) regions were predicted to have a deleterious effect, whereas the other variants were predicted to be neutral. Of the four nucleotide variants in the exonic region, T → G, C → G, G → C and two G → T variants were found to be non-synonymous or missense mutations and one G → T variant was synonymous. The three missense mutations observed in the exon 1 region were found only in the OSCC patients, which was predicted to be deleterious and probably damaging at the second amino acid residue glycine replaced by valine (Gly2Val) (26% of OSCC patients) and the thirteenth amino acid residue phenylalanine replaced with cysteine (Phe13Cys) (6.67% of OSCC patients). The glycine to valine mutation in survivin structure was found to be in the mitochondrial targeting sequence of survivin protein (aa1-10); the mitochondrial targeting sequence imports the survivin protein to mitochondria through its protein-rich N terminus. This survivin is further released from the mitochondria into the cytosol in response to apoptotic stimuli, thus resulting in enhanced anti-apoptotic activity compared to cytoplasmic survivin, and the reason for this is not clear [105,106]. Studies have observed specifically in cancer cells survivin to be additionally detected in mitochondria [106].

In the exon 2 region, the variants observed were all nonsynonymous and of the combined output generated by PredictSNP, three were predicted to have a deleterious effect and two to have a functionally neutral effect. The probable damaging mutation was found in OSCC samples and healthy control samples at alanine replaced with valine at the 41 position of amino acid residue (Ala41Val) and aspartic acid replaced by histidine at the 72 amino acid residue (Asp72His) in the survivin protein structure, which is related to the BIR domain (aa 18–88). The BIR domain is involved in the mitosis of the proliferating cancer cells and in the microtubule dynamics during mitosis [107,108].

Two mutations were identified in the exon 3 region, of which one was a stopgain mutation and one a nonsynonymous mutation. The amino acid mutation was at the 93 position amino acid residue glycine replaced by arginine (Gly93Arg) in the survivin protein structure, and the mutation was predicted to have a benign effect on the protein function and be tolerated, and it was present only in the OSCC samples and was not identified in the tobacco chewers or healthy control group. The amino acid region in the protein structure was involved in dimerization (aa 90–102) of the survivin monomer to form homodimers assisted by the N terminal residue [109]. However, studies have shown survivin protein to reduce caspase activity in both monomeric and homodimeric states; hence, the variant has a neutral effect on the function of the survivin protein [110].

Eleven mutations were identified in the exon 4 region, of which three mutations were synonymous mutations, one was a stopgain mutation, and seven were nonsynonymous mutations. Of the seven nonsynonymous mutations, four mutations at arginine replaced by glycine at the 106 position (Arg106Gly), glutamine replaced with histidine at the 92 position (Gln92His), glutamic acid replaced with aspartic acid at the 107 position amino acid (Glu107Asp), alanine replaced by serine at the 109 position (Ala109Ser), and phenylalanine replaced with leucine at the 93 position amino acid residue (Phe93Leu) were predicted to be tolerated and benign. Two mutations at leucine replaced by phenylalanine at the 96 position amino acid residue (Leu96Phe), predicted to be deleterious and probably damaging, and mutation lysine replaced by isoleucine at the 112 position of amino acid residue (Lys112Ile) in the survivin protein structure were predicted to be tolerated and possibly damaging, respectively.

The mutation at Leu96Phe is in the nuclear export signal region (aa 96–104) and Lys112Ile amino acid is in the inner centromere protein (INCENP) interacting region in the survivin protein [111]. The NES region is critical in the nuclear export of survivin protein and is centrally placed in the survivin protein structure between the BIR domain and the C terminal helix. Survivin is transported out of the nucleus in an exportin-1-dependent manner [112,113]. A study by Temme et al. on newly isolated fibroblasts observed that the ectopically expressed survivin was nuclear but became progressively cytoplasmic with successive passages because of heat shock proteins 90 and phosphorylation at the T34 residue on survivin protein [114,115].

Enhanced INCENP interaction is critical during mitosis, where it forms the chromosomal passenger complex (CPC). The CPC is targeted by the survivin to the centromeres and enables the Aurora B kinase to phosphorylate various proteins that ensure proper alignment of chromosomes during cytokinesis. Experimentally, this region of survivin is shown to be a highly conserved region in eukaryote loss or deletion, which can result in prometaphase defects, cytokinesis failure, and increased apoptosis [116,117,118]. The deleterious mutation at 96 amino acid residue was identified only in OSCC samples; however, the mutation at 112 amino acid was found only in the habitual tobacco chewers.

In the exon 5 region of the BIRC5 gene sequence, we found 11 exonic mutations of which one was a stopgain mutation, 3 were synonymous mutations, and 7 were nonsynonymous mutations. Of the 7 missense mutations, the amino acid residue mutations at arginine replaced with tryptophan at the 86 position (Arg86Trp) and leucine replaced with glutamine at the 100 position (Leu100Gln) were predicted to have deleterious functional effect and be tolerated; glutamine replaced with arginine at the 132 position amino acid mutation (Gln132Arg) was not scored by prediction tools and was identified as an unknown variant. Methionine replaced with isoleucine at position 74 (Met74Ile) and arginine replaced with tryptophan at the 86 amino acid position (Arg86Trp) were in the BIR domain region of the protein, Leu100Gln in the NES region, and Gln132Arg in the microtubule binding region. Variants predicted in the 3′ untranslated region G → T, C → T, A → T, T → G, G → A might be involved in the expression and stability of survivin mRNA. The increased rate of incidence of oral cancer in the study area was strongly linked to epigenetic factors, namely, exposure at an early age to tobacco and alcohol, living in a gold mining area and fluoride-affected belt.

## 5. Conclusions

The study results confirmed that there is an increased concentration of survivin in OSCC tissues compared to other groups. Elevated survivin levels establish suppression of apoptosis in oral squamous cell carcinoma. The absolute monocyte count, LMR, and NLR were found to correlate with tissue survivin levels in OSCC and might serve as useful prognostic biomarkers and generate a link with survivin. Its role in altering the counts of systemic inflammatory cells might be an important implication in the onset of OSCC. The genetic variants identified in the BIRC5 gene of OSCC patients revealed G → T (35%) in the promoter region, the G → C (86.67%) variant in the 5′ untranslated region, in the exon 1 region C → A (33.33%), G → T (26.67%) variants were present in a large number of OSCC patients. The unique variants found only in OSCC patients were T → G in the promotor region; in the exon 3 region G → C; in the exon 4 region C → A, A → G, G → T, T → G, A → C, G → A; in the exon 5 region C → A, G → T, G → C. Observed mutations affecting survivin were Gly2Val, Phe13Cys, Asp72His, Gly93Arg, Leu96Phe, Arg106Gly, Ala109Ser, Met74Ile, and Gly132Arg. These were the important mutations implicated in the exertion of altered biological effect on survivin protein and its interaction with caspases in bringing about the inhibition of caspases in apoptosis during the transformation of normal cells into neoplastic oral squamous cells. These single nucleotide polymorphism/variants can cause an unusual expression of survivin protein and further induce carcinogenesis by interfering with various functional domains of the protein. They can thus be used as a genetic marker to screen for OSCC among tobacco chewers and predict the risk of developing OSCC among habitual tobacco chewers in the presence of these variants in the gene. In future, they may also help us to formulate or modify treatment accordingly, thereby improving the outcome for these patients. This was a preliminary study in the direction of identifying genetic markers in inhibitor of apoptosis protein gene *BIRC5*, specifically in the unexplored coding regions of the gene. However, a limitation is that the study should be conducted in larger cohort with a larger sample size to generate the underlying genetic evidence in OSCC.

## 6. Limitations of the Study

This was a single centric study; the enrolled cases were not followed up for the assessment of the prognostic role of survivin. Capturing survivin mRNA expression was not assayed in the tissues and buccal cells, which might support the information about survivin protein. Pretreatment hematology parameter counts were not included from habitual tobacco chewers and the control group for a pairwise comparison.

## Figures and Tables

**Table 1:** Details of primer pairs used for the amplification of the promoter and exons of the *BIRC5* gene.

Locus	Primer	Sequence	Product Size
Promoter	Forward	GCCTCTCAAAGTGTTGGGATTA	343 bp
	Reverse	GGGCCAGTTCTTGAATGTAGAG	
Exon1	Forward	CCGCCTCTACTCCCAGAA	226 bp
	Reverse	CGCAGCCCTCCAAGAAG	
Exon2	Forward	CTCCCTGCTTTGTCCCCAT	341 bp
	Reverse	GAGGTATCCGTTCACAACAGC	
Exon3	Forward	GCAGTTCTGGTAACGGTGATAG	230 bp
	Reverse	CCGTATTAGCCAAGATGGTCTC	
Exon4	Forward	ATGTCCACAGGGAGAGAGAA	536 bp
	Reverse	GAGAATCACTTGAACCCGAGAG	
Exon5	Forward (Internal Primer)	GAAGCGTCTGGCAGATAC	1070 bp
	Reverse	AGTCTAGGCGGTTGCACTT	

**Table 2:** Mean ± standard deviation of survivin in study groups.

Analytes	Groups	Mean ± SD	95% Confidence Interval for Mean		*p* Value
			Lower Bound	Upper Bound	
Survivin (pg/mL)	Group 1	1670.9 ± 796.21	1466.94	1874.75	<0.001
	Group 2	1096.02 ± 346.17	1008.11	1183.93	
	Group 3	397.5 ± 96.1	373.29	421.69	

**Table 3:** Data on pretreatment hematological parameters in patients with OSCC.

SL. No.	Blood Parameters	Mean ± SD (N = 63)
1	Hemoglobin (gm%)	11.67 ± 1.68
2	RBC (mil/cu.mm)	4.47 ± 0.57
3	Packed Cell Volume (%)	36.14 ± 4.60
4	Mean Corpuscular Volume (fl)	80.85 ± 6.22
5	Mean Corpuscular Hemoglobin Concentration (%)	32.23 ± 1.51
6	Red Cell Distribution Width (%)	15.81 ± 3.04
7	White Blood Cell count (thousands/cu.mm)	7.67 ± 2.75
8	Absolute Neutrophil Count × 10^9^/L	4.38 ± 2.18
9	Absolute Lymphocyte Count × 10^9^/L	1.96 ± 1.02
10	Absolute Monocyte Count × 10^9^/L	0.90 ± 1.76
11	Platelet Count × 10^9^/L	341.1 ± 113.63
12	Neutrophil/Lymphocyte ratio	2.86 ± 2.34
13	Platelet/Lymphocyte Ratio	226.86 ± 144.98
14	Lymphocyte/Monocyte Ratio	3.12 ± 1.50

**Table 4:** Univariate analysis of hematology parameters with survivin levels in OSCC patients.

SL. No.	Survivin pg/mL	Univariate Analysis	
		Pearson Correlation	Sig. (2-Tailed)
1	Hemoglobin	−0.014	0.91
2	RBC	0.023	0.86
3	PCV	0.075	0.56
4	MCV	0.077	0.55
5	MCHC	−0.221	0.08
6	RDW	0.193	0.13
7	WBC	−0.143	0.26
8	ANC	−0.094	0.46
9	ALC	−0.221	0.08
10	AMC	−0.089	0.49
11	PLT	0.037	0.78
12	NLR	0.166	0.19
13	PLR	0.321 *	0.01 *
14	LMR	−0.196	0.12

* *p* < 0.05 statistically significant.

**Table 5:** Univariate analysis of hematology parameters with stage of tumor and lymph node involvement.

Variables	T. Staging	N	Mean	SD	*t* Value	*p* Value	Node Involvement	N	Mean	SD	*t* Value	*p* Value
Hb	T1–T2	13	12	1.50	0.762	0.449	N0	14	11.79	1.63	0.296	0.769
	T3–T4	50	11.6	1.7			N1–N3	49	11.64	1.70		
RBC	T1–T2	13	4.53	0.42	0.455	0.651	N0	14	4.34	0.67	−0.952	0.345
	T3–T4	50	4.45	0.61			N1–N3	49	4.51	0.54		
PCV	T1–T2	13	36.76	3.76	0.542	0.590	N0	14	35.84	4.41	−0.281	0.779
	T3–T4	50	35.98	4.81			N1–N3	49	36.23	4.69		
MCV	T1–T2	13	81.05	7.39	0.129	0.898	N0	14	82.25	4.14	0.957	0.342
	T3–T4	50	80.79	5.96			N1–N3	49	80.45	6.68		
MCHC	T1–T2	13	32.82	1.92	1.610	0.113	N0	14	32.43	2.01	0.554	0.581
	(T3–T4	50	32.08	1.37			N1–N3	49	32.17	1.36		
RDW	T1–T2	13	13.86	2.26	−2.723	0.008 *	N0	14	14.13	1.43	−2.436	0.018 *
	T3–T4	50	16.31	3.03			N1–N3	49	16.29	3.21		
WBC	T1–T2	13	9.35	3.50	2.588	0.012	N0	14	8.10	2.33	0.656	0.514
	T3–T4	50	7.23	2.37			N1–N3	49	7.55	2.87		
ABN	T1–T2	13	5.20	1.82	1.538	0.129	N0	14	4.79	1.85	0.786	0.435
	T3–T4	50	4.17	2.23			N1–N3	49	4.27	2.27		
ABL	T1–T2	13	2.68	1.62	3.063	0.003	N0	14	2.00	0.70	0.190	0.850
	T3–T4	50	1.77	0.70			N1–N3	49	1.94	1.09		
ABM	T1–T2	13	2.20	3.68	1.608	0.134	N0	14	0.64	0.22	−0.610	0.544
	T3–T4	50	0.56	0.17			N1–N3	49	0.97	1.99		
PLT	T1–T2	13	413.23	151.28	2.063	0.057	N0	14	315.77	153.06	−0.945	0.348
	T3–T4	50	322.36	94.83			N1–N3	49	348.35	100.45		
NLR	T1–T2	13	2.67	1.57	−0.328	0.744	N0	14	2.69	1.60	−0.301	0.764
	T3–T4	50	2.91	2.52			N1–N3	49	2.91	2.53		
PLR	T1–T2	13	235.70	174.41	0.245	0.807	N0	14	175.80	101.72	−1.883	0.069
	T3–T4	50	224.56	138.28			N1–N3	49	241.45	152.88		
LMR	T1–T2	13	2.64	1.60	−1.313	0.194	N0	14	3.30	1.07	0.497	0.621
	T3–T4	50	3.25	1.45			N1–N3	49	3.07	1.60		

* *p* < 0.05 statistically significant.

**Table 6:** Multiple linear regression model of hematology parameters with tissue survivin levels.

Dependent Variable: Survivin	**Hematology Parameters**	**Unstandardized Coefficients**		**Standardized Coefficients**	***t* Value**	***p* Value**
		B	Std. Error	Beta		
	(Constant)	12,981.379	12,985.118		1.000	0.324
	Hb	−175.851	221.050	−0.677	−0.796	0.431
	RBC	681.616	645.181	0.897	1.056	0.297
	PCV	17.702	100.027	0.187	0.177	0.860
	MCV	−88.496	132.679	−1.266	−0.667	0.509
	MCHC	−471.015	329.721	−1.637	−1.429	0.161
	RDW	43.961	26.720	0.307	1.645	0.108
	WBC	−205.654	547.736	−1.300	−0.375	0.709
	ANC	312.950	545.156	1.570	0.574	0.569
	ALC	1160.188	722.812	2.711	1.605	0.117
	AMC	−3955.307	1909.181	−16.024	−2.072	0.045 *
	PLT	1.075	1.364	0.281	0.788	0.435
	NLR	226.494	133.105	1.221	1.702	0.097
	PLR	−5.289	2.704	−1.765	−1.956	0.058
	LMR	608.382	260.746	2.089	2.333	0.025 *

* *p* < 0.05 statistically significant.

**Table 7:** Independent *t* test comparing the mean values of tissue survivin with hematological variables in OSCC Patients.

Parameters	Cut-off Values	N	Mean ± Std. Deviation	*t* Value	*p* Value
WBC × 10^9^/L	≥7.9	26	2039.54 ± 394.83	1.080	0.284
	<7.9	37	1919.57 ± 459.11		
Absolute NeutrophilCount × 10^9^/L	≥4.9	21	2067.33 ± 411.62	1.276	0.207
	<4.9	42	1919.95 ± 442.07		
Absolute LymphocyteCount × 10^9^/L	>1.980	20	2020.30 ± 404.10	−0.635	0.528
	≤1.980	43	1945.26 ± 450.54		
Absolute MonocyteCount × 10^9^/L	≥0.50	38	2116.92 ± 380.76	3.645	0.001 *
	<0.50	25	1744.36 ± 420.71		
NLR	≥2.39	21	2241.19 ± 203.90	3.896	0.001 *
	<2.39	42	1833.02 ± 456.42		
LMR	>3.22	33	1717.55 ± 434.75	6.044	0.001 *
	≤3.22	30	2245.77 ± 209.46		
PLR	>110.6	47	2007.19 ± 395.99	−1.197	0.236
	≤110.6	16	1857.13 ± 530.60		

* *p* < 0.05 statistically significant.

**Table 8:** *BIRC5* gene single nucleotide polymorphism/variants based on the analysis of sequence data using the bioinformatics prediction tool PredictSNP2.

Promoter	Group 1 (n = 20)			Group 2 (n = 10)			Group 3 (n = 10)		
Nucleotide Variants	Frequency	%	Tissue Survivin (Average pg/mL)	Frequency	%	Tissue Survivin (Average pg/mL)	Frequency	%	Tissue Survivin (Average pg/mL)
G → T	7	35	2228.58	3	30	1104.12	3	30	443.65
G → C	3	15	2194.29	-	-	-	1	10	437.12
A → T	3	15	2139,39	1	10	1197.3	2	20	428.48
C → T	2	10	2204.96	5	50	1104.12	3	30	427.24
C → G	1	5	2008.01	2	20	1053.68	2	20	428.48
A → G	-	-	-	1	10	1197.3	1	10	424.75
G → A	-	-	-	3	30	1053.68	-	-	-
T → G	1	5	2082.88	-	-	-	-	-	-
**EXON 1**	**Group 1 (n = 30)**			**Group 2 (n = 20)**			**Group 3 (n = 20)**		
**Nucleotide Variants**	**Frequency**	**%**	**Tissue Survivin** **(Average pg/mL)**	**Frequency**	**%**	**Tissue Survivin** **(Average pg/mL)**	**Frequency**	**%**	**Tissue Survivin (average pg/mL)**
G → A	4	13.33	1400.68	-	-	-	2	10	116.35
C → A	10	33.33	1649.3	1	5	1197.3	1	5	180.97
A → C	3	10	1412.43	-	-	-	-	-	-
G → C	26	86.67	1632.77	10	50	1045.56	9	45	398.22
G → T	8	26.67	2015.04	-	-	-	-	-	-
C → G	2	6.67	2110.49	-	-	-	-	-	-
T → G	2	6.67	2427.92	-	-	-	-	-	-
C → T	1	3.33	1565.42	-	-	-	-	-	-
**EXON 2**	**Group 1 (n = 30)**			**Group 2 (n = 20)**			**Group 3 (n = 20)**		
**Nucleotide Variants**	**Frequency**	**%**	**Tissue Survivin (Average pg/mL)**	**Frequency**	**%**	**Tissue Survivin** **(Average pg/mL)**	**Frequency**	**%**	**Tissue Survivin** **(Average pg/mL)**
A → C	-	-	-	1	5	303.97	1	5	432.24
G → C	1	3.33	1074.94	-	-		3	15	432.24
C → T	-	-	-	-	-		1	5	428.65
G → A	1	3.33	1074.94	-	-		2	10	428.65
**EXON 3**	**Group 1 (n = 30)**			**Group 2 (n = 20)**			**Group 3 (n = 20)**		
**Nucleotide Variants**	**Frequency**	**%**	**Tissue Survivin** **(Average pg/mL)**	**Frequency**	**%**	**Tissue Survivin (Average pg/mL)**	**Frequency**	**%**	**Tissue Survivin** **(Average pg/mL)**
C → T	-	-	-	2	10	720.05	-	-	-
G → C	1	3.33	2485.07	-	-		-	-	-
**EXON 4**	**Group 1 (n = 30)**			**Group 2 (n = 20)**			**Group 3 (n = 20)**		
**Nucleotide Variants**	**Frequency**	**%**	**Tissue survivin** **(Average pg/mL)**	**Frequency**	**%**	**Tissue survivin** **(Average pg/mL)**	**Frequency**	**%**	**Tissue survivin** **(Average pg/mL)**
G → C	-	-	-	3	15	1266.55	1	5	422.81
C → T	-	-	-	-	-		1	5	422.81
T → C	1	3.33	1850.2	2	10	1310	-	-	
A → T	2	6.67	1691.5	1	5	1415	2	10	406.12
C → A	1	3.33	2370.77	-	-	-	-	-	-
A → G	2	6.67	2272.69	-	-	-	-	-	-
G → T	3	10	1711.07	-	-	-	-	-	-
T → G	1	3.33	1850.2	-	-	-	-	-	-
A → C	2	6.67	1807.81	-	-	-	-	-	-
G → A	1	3.33	1765.42	-	-	-	-	-	-
T → A	-	-	-	2	10	1157.7	1	5	227.01
**EXON 5**	**Group 1 (n = 30)**			**Group 2 (n = 20)**			**Group 3 (n = 20)**		
**Nucleotide Variants**	**Frequency**	**%**	**Tissue survivin** **(Average pg/mL)**	**Frequency**	**%**	**Tissue survivin** **(Average pg/mL)**	**Frequency**	**%**	**Tissue survivin** **(Average pg/mL)**
A → C	-	-	-	-	-	-	1	5	422.81
C → A	1	3.33	1850.2	-	-	-	-	-	-
T → A	4	13.33	1856.94	2	10	1021.89	2	10	421.03
G → A	2	6.67	1542.09	1	5	628.77	1	5	391.69
C → T	1	3.33	1244.67	1	5	712.23	2	10	327.18
G → T	2	6.67	1542.09	-	-	-	-	-	-
T → G	-	-	-	1	5	1415	-	-	-
G → C	1	3.33	1850.2	-	-	-	-	-	-
A → T	1	3.33	1244.67	-	-	-	1	5	441.29

Overall Mean ± SD (n = 63) Tissue Survivin (pg/mL) Group 1 = 1670.9 ± 796.21, Group 2 = 1096.02 ± 346.17, Group 3 = 397.5 ± 96.1.

## Data Availability

The data presented in this study are available on request from the corresponding author.
